# Extended cost-effectiveness analysis in global health systems: a scoping review of geographic gaps and research trends

**DOI:** 10.1186/s12962-026-00791-4

**Published:** 2026-06-28

**Authors:** Saman Najafi, Mahmoud Nekoei Moghadam, Mohammad Reza Amiresmaili, Mohsen Barouni, Ali Akbar Haqhdoost, Mohammad Jafari Sirizi, Reza Goudarzi

**Affiliations:** 1 Department of Health Management and Policy and Economic, Faculty of Management and Medical Information Sciences, Kerman University of Medical Sciences, Kerman, Iran; 2 Health in Disasters and Emergencies Research Center, Institute for Futures Studies in Health, Kerman University of Medical Sciences, Kerman, Iran; 3 Health Services Management Research Center, Institute for Futures Studies in Health, Kerman University of Medical Sciences, Kerman, Iran; 4 HIV/STI Surveillance Research Center and WHO Collaborating Center for HIV Surveillance, Institute for Futures Studies in Health, Kerman University of Medical Sciences, Kerman, Iran; 5 Iran Insurance Health Kerman Office, Kerman, Iran

**Keywords:** Extended cost-effectiveness analysis (ECEA), Financial risk protection (FRP), Health equity, Public health policy, Resource allocation, Scoping review, Health economics

## Abstract

**Background:**

Extended Cost-Effectiveness Analysis (ECEA) extends conventional cost-effectiveness analysis by incorporating financial risk protection (FRP) and examining the distribution of health and economic outcomes across socioeconomic groups. This scoping review aimed to map the application of ECEA in health-sector studies, identify methodological patterns, and explore geographic and thematic research gaps.

**Methods:**

A scoping review was conducted in accordance with PRISMA 2020 guidelines. Searches were performed on 29 October 2025 in PubMed, Scopus, Web of Science, ProQuest, and Google Scholar. Studies published between 2000 and 2025 that applied ECEA to evaluate health interventions were eligible for inclusion. Data were extracted on intervention characteristics, analytical approaches, equity measures, and FRP outcomes and were synthesized narratively.

**Results:**

A total of 1,955 records were identified, of which 19 studies met the inclusion criteria. Most studies were conducted in low- and middle-income countries, particularly in Asia and Africa. The included studies primarily evaluated vaccination programs, taxation policies on harmful products, publicly financed health services, and maternal and child health interventions. Financial Risk Protection (FRP) outcomes were most commonly reported as reductions in out-of-pocket expenditures, catastrophic health expenditures averted, and poverty cases averted. Across studies, ECEA was predominantly used to assess the distribution of health and financial outcomes across socioeconomic groups, with outcomes commonly reported by income quintiles.

**Conclusion:**

ECEA applications remain limited and are predominantly concentrated in low- and middle-income countries, particularly within preventive and fiscal health interventions. The reviewed evidence suggests that ECEA provides a useful framework for assessing both health outcomes and financial risk protection across socioeconomic groups. Future research should expand ECEA applications to underrepresented settings and promote greater methodological standardization of equity and FRP measures.

**Supplementary Information:**

The online version contains supplementary material available at https://doi.org/10.1186/s12962-026-00791-4.

## Introduction

Health is fundamental to human development, influencing individual well-being, productivity, and broader socioeconomic progress. Investments in preventive care, public health programs, and equitable access to services have contributed to improvements in population health, particularly in low- and middle-income countries (LMICs), as demonstrated in recent reports from the WHO and the World Bank [1–4]. This growing recognition of health’s economic and social value underscores the need for tools such as Extended Cost-Effectiveness Analysis (ECEA) that integrate efficiency with equity considerations.

Traditional cost-effectiveness analysis (CEA) remains an essential tool for comparing the efficiency of health interventions; however, it does not capture how health and financial outcomes are distributed across population groups with differing levels of vulnerability. This limitation is critical for policymakers in resource-constrained settings, where equity considerations are central to health system performance. Extended Cost-Effectiveness Analysis (ECEA) was developed to address this gap by jointly quantifying health gains and financial risk protection (FRP), thereby providing policymakers with metrics that reflect both efficiency and equity. ECEA evaluates distributional outcomes across socioeconomic subgroups, including impacts on out-of-pocket (OOP) expenditures, poverty reduction, and catastrophic health spending. The approach has been applied in various domains such as vaccination strategies, chronic disease management, and public health regulations, offering insights for designing more equitable health interventions [1, 5–7]. ECEA further advances equity-focused economic evaluation by explicitly embedding distributional welfare principles—such as equity weights and concentration-based measures into economic assessment frameworks, thereby linking population health gains with household-level financial protection. This aligns with broader conceptual developments in equity informative economic evaluation [7, 8] .

A growing body of ECEA studies has emerged across diverse settings, including China [6, 11,], Iran [12], the Philippines [13], South Africa [1, 14], Mexico [15], India [5, 16–19], Ethiopia [16, 20–24], Vietnam [25], Lebanon [26] and Nigeria [27]. Despite this expanding evidence base, methodological practices remain heterogeneous, and there is limited synthesis of how ECEA has been operationalized across different health systems.

Accordingly, this scoping review aims to systematically map the global applications of ECEA within the health sector, describe methodological variations in its implementation, and identify gaps in current evidence to inform future methodological refinement and equity-focused policy development.

ECEA serves as an analytical framework that evaluates both the health and financial impacts of interventions across population subgroups. It enables assessment of broader outcomes such as financial risk protection (FRP), poverty reduction, and mitigation of catastrophic health expenditures. This approach has been applied in diverse contexts including vaccination programs, chronic disease management, and public health policies providing valuable insights for policymakers aiming to enhance both efficiency and equity in health resource allocation [9]. In addition, ECEA is conceptually linked to advances in distributional economic evaluation, including the Distributional Cost-Effectiveness Analysis (DCEA) framework developed by Cookson [7] et al. (2017), which explicitly integrates equity considerations and social welfare functions into economic evaluation. Referencing this literature further situates ECEA within the broader field of equity-informative health economics.

Beyond its practical applications, ECEA is grounded in welfare economics, extending conventional CEA by integrating principles of distributive justice and social protection. Seminal contributions by Verguet et al. (2015) and Watkins et al. (2017) demonstrate how ECEA links economic evaluation with equity-oriented decision-making by quantifying the distribution of both health benefits and financial outcomes across income groups. This theoretical expansion enables a more complete assessment of how interventions contribute to overall social welfare an especially important consideration in contexts marked by socioeconomic inequalities [9, 10].

## Methods

### Information sources and search strategy

This scoping review was conducted on 29 October 2025, in accordance with the PRISMA 2020 framework for Scoping Reviews [11]. A scoping review methodology was selected because the overarching purpose of this study was to comprehensively map how Extended Cost-Effectiveness Analysis (ECEA) has been conceptualized, operationalized, and methodologically applied within the health sector. ECEA studies span a wide range of interventions (preventive, curative, fiscal), analytic structures, distributional metrics, and outcome frameworks, creating substantial methodological heterogeneity that precludes aggregative synthesis.

By using a scoping approach, we were able to:1- systematically chart methodological choices across studies (equity metrics, FRP computation, quintile stratification, perspective selection). 2- identify inconsistencies and underreported elements (e.g., uncertainty analysis, discounting practices, reporting of currency year and equity measures). 3-highlight conceptual and methodological gaps in the application of ECEA. 4-map emerging trends to guide future methodological development and policy-relevant research. Thus, the scoping review framework aligns directly with the study’s objective of synthesizing methodological diversity rather than comparing intervention-specific effectiveness.

The study included all published articles in English related to ECEA in the health sector, covering the period from 2000 to 29 October 2025. The timeframe (2000–2025) was selected because ECEA as a distinct evaluation framework emerged in the early 2000s and was formalized in the tutorial by Verguet et al. (2016) and further elaborated in the DCP3 volume on health policy assessment. The search strategy combined relevant keywords and Medical Subject Headings (MeSH) terms to ensure comprehensive coverage of the literature [6].

To address the research objective, a comprehensive search was conducted across major health and multidisciplinary databases, including PubMed, Scopus, Web of Science, and ProQuest. These databases were selected to ensure broad coverage of peer-reviewed literature, while ProQuest was also used to capture relevant grey literature such as dissertations and institutional reports. In addition to these sources, Google Scholar was systematically searched to identify non-indexed materials, including research reports, working papers, and policy documents. Grey literature sources specifically included World Health Organization (WHO) technical reports, World Bank policy briefs, and national health financing documents retrieved through ProQuest and Google Scholar. Advanced search functions and consistent combinations of keywords and Medical Subject Headings (MeSH) were used across databases, applying Boolean operators (“AND,” “OR”) to ensure both sensitivity and precision. Manual reference checking of included articles and citation chaining were performed to ensure completeness of the search process.

The search strategy employed a comprehensive combination of keywords and MeSH terms, including “extended cost-effectiveness analysis”, “ECEA”, “distributional cost-effectiveness analysis (DCEA)”, “equity-informative CEA”, “financial risk protection”, “poverty impact”, “equity analysis”, “health care”, and “health system”. Additional synonymous and conceptually related terms were incorporated to enhance the sensitivity of the search. Boolean operators (**AND**, **OR**) were applied to combine terms systematically across all databases.

A detailed description of the full search syntax, including database-specific keyword combinations and Boolean logic, is provided in Supplementary File 1 to ensure transparency and reproducibility of the search process.

### Study selection and data collection process

Under the PRISMA 2020 framework, the selection process began with the screening of titles and abstracts of the retrieved articles. This initial screening was performed independently by two reviewers to minimize potential bias. Articles deemed relevant by at least one reviewer progressed to the next phase. In the subsequent stage, the full texts of the selected articles were meticulously reviewed against predefined inclusion and exclusion criteria. Each reviewer independently assessed the eligibility of the articles, determining whether to include or exclude them. In instances of disagreement between the two reviewers a third reviewer) R.G) was consulted to reach a consensus. All decisions made during each phase of the process were systematically documented, including the use of selection forms and a detailed rationale for inclusion or exclusion. Finally, the overall methodology and results of the selection process were summarized and visually presented in a PRISMA flow diagram, ensuring clarity and transparency in reporting.

### Inclusion and exclusion criteria

The inclusion criteria for this review encompassed research studies and original articles, as well as grey literature pertaining to Extended Cost Effectiveness Analysis (ECEA) and health interventions conducted in various countries around the world. Only articles published in English Language between 2000 and 2025 were eligible for consideration. Exclusion criteria were applied to articles that were classified as clinical trials, case studies, letters to the editor, editorial pieces, comments, book chapters, or conference proceedings.

No geographic or income level restrictions were applied during the study selection process. However, the evidence retrieved through the systematic search was overwhelmingly concentrated in low- and middle-income countries (LMICs), reflecting the fact that ECEA has been predominantly developed and applied in settings where financial risk protection, poverty reduction, and distributional equity are central policy priorities.

A small number of studies originating from high-income countries (HICs) were identified at the initial search stage, but these did not meet the eligibility criteria required for final inclusion particularly with respect to the explicit use of the ECEA framework, the reporting of distributional outcomes by income group, and the definition or calculation of financial risk protection metrics. Consequently, the focus on LMIC evidence in this review reflects the current structure of the published literature rather than a predefined restriction imposed by the review design.

### Data extraction and analysis

A systematic approach to data extraction and analysis was maintained through the adoption of a structured methodology. Predefined criteria ensured the inclusion of studies directly relevant to the research questions while excluding those of lower quality or unrelated to the topic. To streamline data collection and document the bibliographic details of each study, a standardized data extraction form was created using Microsoft Excel and Word 2019.

The extracted data included essential elements such as authors’ names, publication year, time horizon, population size, study perspective, type of intervention, Alternative intervention, costs, measured outcomes, cost effectiveness levels across quintiles, and the Financial Risk Protection (FRP) index. To enhance accuracy, two reviewers independently conducted data extraction, with any discrepancies resolved through consultation with a third reviewer to achieve consensus.

Extracted data were synthesized using both narrative and thematic approaches, guided by the framework proposed by Arksey and O’Malley (2005) and refined by Levac et al. (2010) for scoping reviews. Thematic synthesis was applied to identify recurring methodological patterns, application areas, and outcome indicators across studies. The findings were descriptively summarized and mapped according to study characteristics, intervention types, and equity-related outcomes, thereby providing a structured overview of global ECEA research trends [12, 13].

All extracted cost data were recorded in U.S. dollars (USD), using the units reported in the original studies; PPP-adjusted estimates were retained when provided.

### Equity metrics and financial risk protection measures

To capture the distributional dimensions central to Extended Cost-Effectiveness Analysis (ECEA), this review systematically extracted and categorized the equity metrics applied across the included studies. Equity was primarily operationalized through income-quintile disaggregation, whereby health and financial outcomes were reported separately for each socioeconomic group. Several studies further incorporated formal equity measures such as concentration indices, progressivity analyses, and equity weighting frameworks to quantify distributional patterns more rigorously.

To ensure methodological consistency, all Financial Risk Protection (FRP) indicators were classified into three standardized categories: (1) out-of-pocket (OOP) expenditures averted, (2) catastrophic health expenditure (CHE) cases averted, and (3) poverty cases averted. When a study reported FRP outcomes without specifying the underlying calculation method or threshold definition, the metric was coded as Unclear. This harmonized coding approach enabled systematic comparison of heterogeneous FRP measures across studies.

FRP was defined in accordance with the original ECEA framework proposed by Verguet et al., which conceptualizes financial protection as the extent to which an intervention reduces households’ exposure to medical and economic hardship. Because definitions and computational methods for FRP varied considerably across the literature, the standardized classification described above was applied consistently [14]. This approach ensured transparency, comparability, and alignment with best practices in scoping review methodology.

For transparency, the specific Financial Risk Protection (FRP) computation method used in each study including OOP reduction formulas, CHE thresholds (e.g., OOP > 10% of total consumption or > 25% of non-food expenditure), and poverty-line definitions (e.g., $1.90/day or national poverty lines) is provided in Supplementary Table S1. Studies that did not report an explicit FRP calculation method were coded as “Not specified.”

### Quality appraisal

To enhance methodological rigor and transparency, we conducted a structured quality appraisal of all included studies using a modified version of the **Consensus Health Economic Criteria (CHEC) checklist**, a well-established tool for assessing the methodological quality of economic evaluations. The CHEC-list consists of **20 items** covering key components of study design, costing, outcome measurement, validity, reporting standards, and transparency. For this review, the tool was adapted to ensure suitability for Extended Cost-Effectiveness Analysis (ECEA), which incorporates both health and financial risk protection outcomes.

The modified 20-item CHEC checklist used in this review assessed whether each study: (1) clearly stated its objective; (2) defined the study population and/or decision context; (3) specified the analytical perspective; (4) identified relevant comparators; (5) reported the time horizon; (6) identified and described all cost components; (7) identified and described all outcome measures; (8) applied an appropriate economic model; (9) used valid and appropriate cost data; (10) used valid and appropriate outcome data; (11) transparently reported all data sources; (12) clearly described the methods for costing and valuation; (13) included sensitivity or uncertainty analyses; (14) specified relevant payers or stakeholders; (15) addressed potential sources of bias; (16) reported discount rates when applicable; (17) reported currency units and base-year adjustments; (18) discussed generalizability or transferability; (19) disclosed conflicts of interest and funding sources; and (20) presented conclusions consistent with the reported results.

Two reviewers (S.N. and R.G.) independently assessed each included study against the 20-item checklist. Any discrepancies were resolved through consensus with a third reviewer (M.A.). Each criterion was coded as **“Yes**,**” “No**,**” or “Unclear**,**”** with “Unclear” assigned when reporting was insufficient to determine compliance.

In accordance with scoping review methodology, the quality appraisal was used **descriptively** and **not as an exclusion criterion**. Instead, it provides an overview of methodological strengths and weaknesses across the ECEA literature. A summary of appraisal outcomes is presented in CHEC-List Quality Appraisal Table.

### Data synthesis and handling heterogeneity

Extracted data were synthesized using a narrative and thematic approach, consistent with scoping-review best practices. To structure the synthesis, we grouped studies by intervention type: (i) preventive interventions (e.g., vaccination programs), (ii) fiscal/taxation policies (e.g., tobacco or sugar-sweetened beverage taxes), (iii) health-financing strategies (e.g., universal public finance or subsidies), and (iv) clinical or service-delivery interventions. Within each group, we compared key methodological features (analytical perspective, time horizon, model structure, and cost components), distributional approaches, and the methods used to quantify Financial Risk Protection (FRP).

Given the substantial heterogeneity in study designs, outcomes, and model structures, findings were reported descriptively rather than pooled statistically. We conducted pre-specified subgroup comparisons across several dimensions: intervention category (e.g., taxation vs. vaccination), FRP metric (out-of-pocket [OOP] expenditures averted, catastrophic health expenditure [CHE] cases prevented, poverty cases averted), analytical perspective (societal vs. health-system), and time horizon (short-term vs. lifetime). The results of these subgroup comparisons are summarized in Table 3 (main results) and Table 4 (meta-narrative synthesis).

In addition, we adopted a meta-narrative perspective to explore how distinct research traditions within the ECEA literature conceptualize and operationalize equity metrics and FRP. This involved identifying dominant conceptual strands, mapping the terminology and metrics used within each strand, and contrasting methodological choices and reported outcomes across strands. This combined narrative, thematic, and meta-narrative approach enabled us to highlight methodological patterns and areas of inconsistency that warrant future harmonization.

## Results

### Literature search results

This scoping review aims to evaluate studies employing the Extended Cost-Effectiveness Analysis (ECEA) framework to assess a broad range of health interventions, including vaccination programs, taxation of harmful products, publicly financed essential health services, and interventions addressing equity and financial risk protection. The updated and expanded search strategy incorporated a comprehensive set of keywords such as “extended cost-effectiveness analysis,” “ECEA,” “distributional cost-effectiveness analysis (DCEA),” “equity-informative CEA,” “financial risk protection,” and “poverty impact” to ensure maximal sensitivity and capture the full spectrum of equity-oriented economic evaluations.

**Fig. 1:**
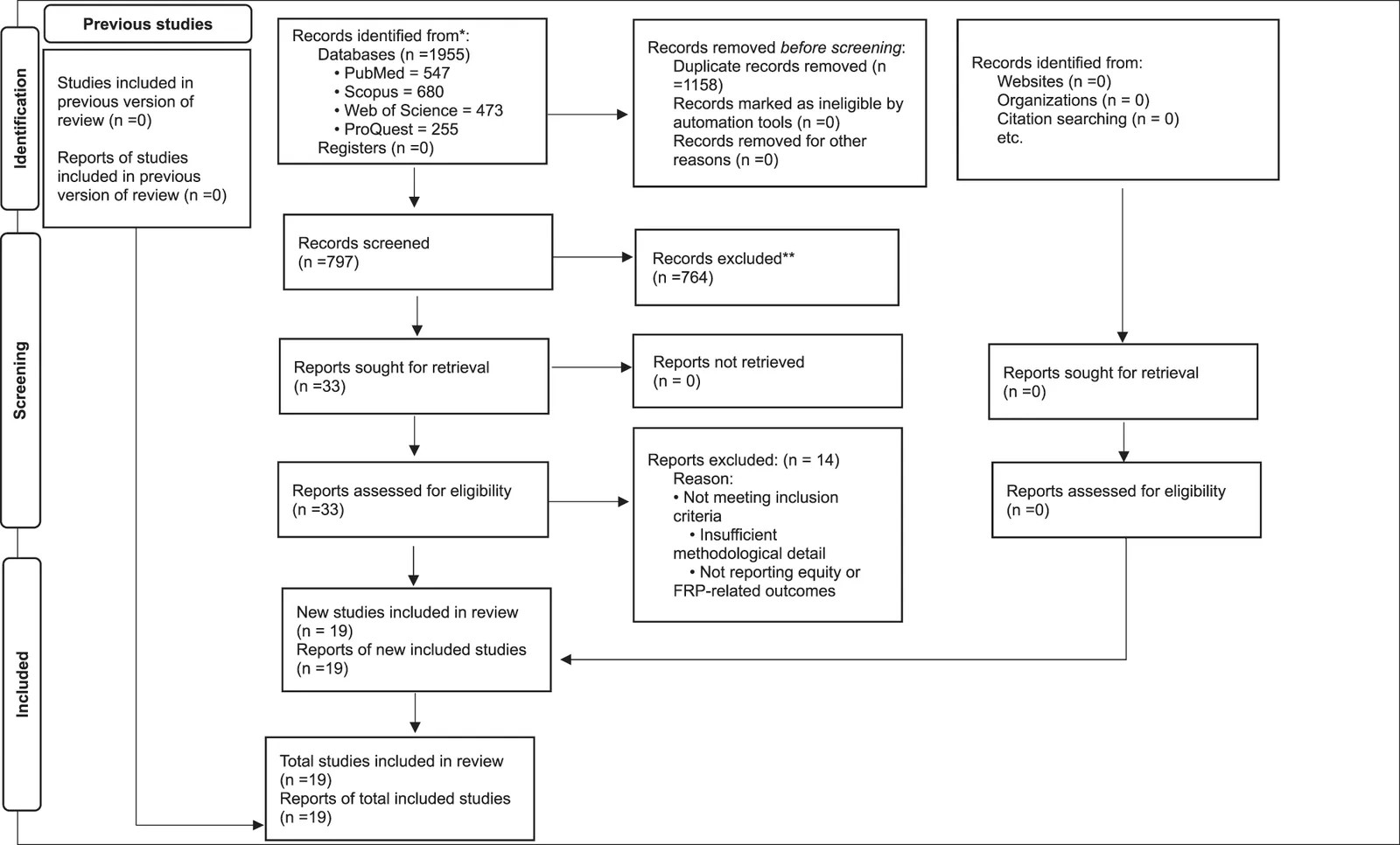
PRISMA 2020 flow diagram for study selection

The database search identified a total of 1,955 records, including 547 from PubMed, 680 from Scopus, 473 from Web of Science, 255 from ProQuest, and one additional record identified through manual searching. After removing 1,158 duplicates, 797 unique records remained for title and abstract screening. Of these, 764 documents were excluded for not meeting the inclusion criteria. A full-text review was conducted for 33 articles, leading to the exclusion of 14 studies that did not satisfy the eligibility criteria. Ultimately, 19 studies met the inclusion criteria and were included in the final synthesis. The study selection process is illustrated in Fig. 1, following the PRISMA 2020 guidelines [11].

### Characteristics of the studies

A total of 19 studies were included in this scoping review. The majority (14/19) were conducted in low- and middle-income countries (LMICs), underscoring the importance of ECEA in resource-constrained settings [12].

Common intervention types included vaccination programs (7 studies), taxation policies on harmful products such as tobacco and sugary beverages (5 studies), public financing or subsidy-based interventions (3 studies), and other context-specific health system interventions (4 studies), such as maternal and child health packages and chronic disease management. The studies represented diverse geographical settings, including South Africa, the Philippines, Mexico, Iran, Armenia, China, Lebanon, and Vietnam [1, 2, 6, 9, 14–24].

Overall, most studies adopted a societal or public-sector perspective, assessing both government and household costs, while a few focused on specific population subgroups (e.g., smokers, children, or adolescents) [8, 15, 18, 19, 25].The primary outcomes measured were health gains (e.g., deaths averted, DALYs or QALYs gained) and financial protection effects, particularly reductions in out-of-pocket expenditures and catastrophic health spending [9].

Collectively, these studies demonstrate that ECEA has been most commonly applied to interventions offering dual benefits improving health outcomes while reducing financial burdens among lower-income groups. The detailed characteristics of all included studies are presented in Table 1.

**Table 1:** Characteristics of included studies

Row	Author (Study Year (	Country	Time Horizon	Size of population	perspective	Policy	Comparison	Costs	Outcome measured	Which quintile is cost-effective	FRP INDEX	Source
1	Verguet et al. (2013)	India and Ethiopia	five years	Hypothetical cohort of 1,000,000 births followed over five years	Societal perspective; considering costs borne by governments and households	Publicly financed rotavirus vaccination program	Pre-vaccination scenario where individuals pay out of pocket for rotavirus treatment	• **Vaccine price**: $5.00 per 2-dose course (with GAVI subsidy: $0.40) • Incremental vaccination system cost: $0.5 per 2-dose course • Out-of-pocket expenses for rotavirus treatment differed across income groups and treatment types (inpatient vs. outpatient).	• Rotavirus deaths averted • Household expenditures averted • Financial risk protection (FRP) • Distributional consequences across wealth strata	Most cost-effective for the lowest-income populations.	money-metric value	**(Verguet et al.**,** 2013)**
2	Verguet, S., et al.)2015(	India	NA	1,000,000	public sector	Universal Public Finance (UPF) of TB treatment, primarily through the Directly Observed Treatment, Short-course (DOTS) strategy.	The study compares the outcomes with and without UPF for TB treatment) Borrowing and asset sales. (	• Cost per course of treatment under DOTS: approximately $100 (2011 USD). • Non-DOTS treatment: approximately $50 (2011 USD). • Private expenditures and crowding out effects are considered.	• Tuberculosis deaths averted • private expenditures crowded out • Insurance value.	The study concludes that the health gains and financial protection mainly benefit the lower-income quintiles.	Insurance value	**(Verguet et al.**,** 2015b)**
3	Verguet, s et al(2015)	China	50 years	male population, with the size of the male population in China being 677 million	Societal	A 50% increase in tobacco price through excise tax	before and after the tax increase	• Household expenditures on tobacco • health care expenditures on tobacco-related diseases	• Years of life gained • additional tax revenues • net financial consequences for households • financial risk protection	The health and financial benefits would disproportionately advantage the lower-income population quintiles.	money-metric value of insurance	**(Verguet et al.**,** 2015a)**
4	Saxena, A., et al.)2019(	South Africa		**NA** (The study considers the entire population across income quintiles.)	**NA**	Implementation of a 10% tax on sugar-sweetened beverages.	Do nothing )The study compares outcomes with and without the SSB tax(	• Out-of-Pocket payment • Indirect Costs (presenteeism and absenteeism)	• the number of premature deaths averted due to T2DM • healthcare savings • tax revenues • financial risk protection (FRP)through reduced out-of-pocket expenses averted catastrophic expenditures and poverty cases	The third- and fourth-income quintiles experience the most significant health benefits (in terms of deaths averted), while the lowest two quintiles carry the smallest tax burden.	averted out-of-pocket costs and reduced cases of catastrophic health expenditures.	**(Saxena et al.**,** 2019b)**
5	Saxena, A., et al.)2019(	Philippines		98.2 million (as of 2013)		Implementation of a 6 Philippine pesos per liter excise tax on sweetened beverages	No tax (baseline consumption and health outcomes prior to the tax implementation).	• Health care costs associated with inpatient settings	• Number of premature deaths averted due to type 2 diabetes mellitus • reduction in out-of-pocket payments • financial risk protection (e.g., cases of catastrophic expenditure averted).	The fourth and fifth (highest) income quintiles are expected to experience the greatest health benefits.	catastrophic health expenditure (CHE): The study estimated that 13,890 cases of catastrophic expenditure could be averted.	**(Saxena et al.**,** 2019a)**
6	-Saenz-de-Miera B.)2022(	Mexico	Life time	The study evaluates impacts on a population where approximately 1.5 million smokers would potentially quit smoking.	National	one-peso increase in the specific component of the tobacco excise tax (from 0.4944 to 1.4944 pesos per cigarette)	Current scenario with existing tax rates	The study mentions averted treatment costs amounting to 44.6 billion pesos and additional tax revenues of 16.2 billion pesos	• Life-years gained • Smoking attributable deaths averted • Treatment costs averted • Number of persons avoiding poverty • catastrophic health expenditures	• The study shows that the lowest income quintile gains three times more life years than the highest, particularly in the South region.	• Poverty • avoid catastrophic health expenditures. 251,000 individuals would avoid poverty, and 563,900 would avoid catastrophic health expenditures	**(Saenz-de-Miera et al.**,** 2022)**
7	Raykar, N, et all)2016(	India	Single year (Static model)	Cohort of 1 million, evenly distributed into 200,000 individuals per quintile	NA	Universal public finance (UPF) of schizophrenia treatment with 80% target coverage	Current scenario with varying coverage rates across income quintiles (30% in poorest to 50% in richest)	• Primary health center (antipsychotic medication, basic and intensive psychosocial treatment) and hospital. • Outpatient visits for short term inpatients • Inpatient treatment- psychiatric unit-short term • Inpatient treatment- residential unit-long term) • Drug **(**Chlorpromazine • Haloperidol, Risperidone, Fluphenazine, Biperiden) • Other (Lab tests) • Total cost per case (2012 USD/year): 177.42	• -Disability-adjusted life years (DALYs) averted • Out-of-pocket expenditures averted • Value of insurance (financial risk protection)	The study emphasizes that the health benefits are primarily experienced by the poorest quintiles, with the most significant financial risk protection observed in the poorest quintile.	• UPF averts out-of-pocket expenditures • Value of insurance is highest for the poorest quintile and declines with income	**(Raykar et al.**,** 2016)**
8	Raei, B.et al)2021(	Iran	60 years	The study focused on male Iranian smokers aged 15 and above (31562000)	societal	A hypothetical 75% increase in cigarette prices through taxation	Do nothing	• Evaluated averted out-of-pocket costs • health system expenditures • additional tax revenues generated from the excise tax.	• Premature deaths averted • years of life gained • expenditures on tobacco-related disease treatment averted • total out-of-pocket savings • additional tax revenues • change in annual expenditures on cigarettes • poverty cases averted • number of cases of catastrophic expenditure averted.	Cost-effectiveness varied by expenditure quintile with Quintiles I (poorest) and II showing significant benefits.	averted out-of-pocket expenditures	**(Raei et al.**,** 2021)**
9	Postolovska et al.)2018)	Armenia	NA	The study considers all Armenian men alive in 2015, amounting to 1,419,370 males.	NA	Significant increase in tobacco excise tax, resulting in an approximate 145% increase in tobacco retail price	The study compares the scenarios before and after the tobacco tax increase.	• Price per pack of cigarettes (USD 2014) before tax increase • Tobacco-related disease treatment costs (USD 2014).	• Premature deaths averted (in 1000s) • Out-of-pocket expenditures related to tobacco-related disease treatment averted (million USD) • Government savings related to tobacco related disease treatment averted (million USD) • Poverty cases averted (in 1000s) • Cases of catastrophic health expenditures (> 10% of consumption) averted (in 1000s)	The study highlights that the benefits, including averted poverty and catastrophic expenditures, accrue significantly to the lower quintiles	cases of medical impoverishment averted	**(Postolovska et al.**,** 2018)**
10	Pecenka CJ et al. (2015)	Ethiopia		Approximately 92 million (total population of Ethiopia)	Policymakers considering public financing for health interventions	Universal public finance (UPF) of diarrheal treatment and rotavirus vaccination	Baseline: 32% treatment-seeking and no rotavirus vaccination vs. a 20-percentage point increase in treatment-seeking and implementation of rotavirus vaccination.	• Estimated government cost for UPF of diarrheal treatment: approximately US$100 million annually. • Estimated government cost for UPF of both diarrheal treatment and rotavirus vaccination: approximately US$93 million annually.	• Deaths averted • Private expenditures averted • Life-years gained	The health benefits primarily accrue to the poorer quintiles, while financial benefits tend to favor wealthier populations.	reduction in out-of-pocket expenditures.	**(Pecenka et al.**,** 2015)**
11	Mori et al. (2017)	Zambia	NA	Approximately 4900 girls (from 157 clusters).	societal	Comprehensive adolescent pregnancy prevention program, including economic support with or without community dialogue	Control group receiving standard school services and writing materials	• Program implementation • healthcare costs associated with adolescent pregnancy • educational costs	• Net benefits from the program (including health-related and non-health-related outcomes) • averted healthcare costs • increased school completion rates	Not explicitly stated, but the program aims to benefit lower-income quintiles significantly.	reducing out-of-pocket expenditures and catastrophic health expenditures associated with adolescent pregnancies.	**(Mori et al.**,** 2017)**
12	Mao et al. (2023)	Nigeria	2019 to 2030	Approximately 191 million (total population of Nigeria)	Societal	Public financing of 18 essential maternal, newborn, and child health (MNCH) services with zero out-of-pocket costs to patients at the point of care.	-Status quo (coverage expansion as observed from 2013–2018) Uniform scale-up (5% annual coverage increase) Pro-poor scale-up (annual coverage increase of 10%, 8%, 6%, 4%, and 2% from the poorest to wealthiest quintiles).	The study estimated the costs of implementing the interventions, but specific cost figures were not provided in the excerpt	• Deaths averted (under-5 and maternal) • Life-years gained • Private expenditure averted • Cases of catastrophic health expenditure averted	The policy would be highly cost-effective overall, with greater benefits accruing to the poorer quintiles.	The policy would avert approximately US $1.8 billion in private expenditure 3266 cases of catastrophic health expenditure.	**(Mao et al.**,** 2023)**
13	Johansson et al. (2016)	Ethiopia	The interventions are implemented over a 10-year period, with health benefits counted over a lifetime.	Not explicitly provided, but the study considers the entire Ethiopian population divided into five income quintiles.	Societal	**Epilepsy**: 75% coverage with phenobarbital **Depression**: 30% coverage with fluoxetine, cognitive therapy, and proactive case management **Bipolar disorder**: 50% coverage with valproate and psychosocial therapy **Schizophrenia**: 75% coverage with haloperidol plus psychosocial treatment	Interventions versus no scale-up	• Total expected cost: US$177 million • Schizophrenia: US$31 million • Bipolar disorder: US$44 million • Depression: US$65 million • Epilepsy: US$37 million	• Healthy-life-years (HALYs) gained • Household out-of-pocket (OOP) expenditures averted • Expected financial risk protection (FRP) • Productivity impact	The health benefits would be concentrated among the poorest groups for all interventions.	money-metric value of insurance	**(Johansson et al.**,** 2017)**
14	Fraser et al. (2024)	South Africa	45 years	The study simulated a population of approximately 12 million individuals aged between 25 to 69 years in the lowest two income quintiles in South Africa.	Health system	A conditional cash transfer (CCT) program with three strategies: Covering diagnosis services only Covering treatment services only Covering both diagnosis and treatment services	Compared the three CCT strategies to a ‘no program’ scenario.	• Diagnosis only: $110 million total, $18 per person eligible • Treatment only: $67 million total, $11 per person eligible • Diagnosis + Treatment: $69 million total, $12 per person eligible	• Disability-adjusted life years (DALYs) averted • Financial risk protection (FRP) benefits measured as catastrophic health expenditure (CHE) cases averted	The CCT program was found to be most cost-effective for the bottom two income quintiles, i.e., the poorest 40% of the population.	catastrophic health expenditure (CHE) cases averted	**(Fraser et al.**,** 2024)**
15	Essue, B. M. et al. (2019)	Vietnam		Incident cohort from a population of 20,051,000 (Vietnamese population aged over 50).	Societal perspective	**Programme A**: Eliminating medical out-of-pocket costs for small incision cataract surgery. **Programme B**: Program A plus a voucher programmed covering non-medical out-of-pocket costs.	Current status quo where patients pay all medical and non-medical out-of-pocket costs.	• Incremental cost per year of Programme A: $833,396. • Incremental cost per year of Programme B: $1,641,835.	• Disability-adjusted life years (DALYs) averted • cases of catastrophic health expenditure (CHE) averted • cases of medical impoverishment averted.	Programme B would avert the most CHE cases overall and have a greater share of benefits for females, particularly in the poorest income quintile.	• CHE • impoverishing expenditures	**(Essue et al.**,** 2020)**
16	J. Driessen et al. (2015)	Ethiopia		Annual birth cohorts of 2,970,200 over ten years	Societal	**Three measles vaccination programs**: Incremental increases in MCV1 (P1) Incremental increases in MCV1 supplemented by financial incentives (P2) Supplemental immunization activity (SIA) (P3)	Different vaccination strategies compared in terms of costs and outcomes	• Inpatient visit cost • Outpatient visit cost • Inpatient visit cost paid out-of-pocket • Outpatient visit cost paid out-of-pocket • Transport costs • MCV1 cost per child immunized • SIA cost per child immunized • Amount of financial incentive • Administrative costs for P2(10% of total vaccination costs)	• Measles deaths averted • Household expenditures averted • Financial risk protection (FRP)	Routine immunization was cost-effective in the first- and second-income quintiles. Routine immunization with financial incentives was cost-effective in the fourth- and fifth-income quintiles. Supplemental immunization activities were cost-effective in the first- and second-income quintiles.	FRP afforded by the program measured by an imputed percent change in individual income after implementation of either vaccination program	**(Driessen et al.**,** 2015)**
17	Dixit et al. (2023)	India	Lifetime	Hypothetical cohort of 5-year-old healthy children; 702 patients in a registry	societal	Primary, secondary, and tertiary prevention strategies, both in isolation and in combinations	Routine care scenario comprising a mix of interventions at their real-world coverage	• Outpatient consultation at primary health centers, community health centers, and district hospitals • Costs for medical consultation, diagnostic tests, and treatment for patients diagnosed with rheumatic fever • Costs for managing complications such as congestive heart failure, stroke, and infective endocarditis • Costs for hospitalization, surgical procedures, and long-term care • Out-of-pocket expenditure (OOPE) by patients on medications, consultations, diagnostic tests, and treatments	• Number of rheumatic fever and rheumatic heart disease cases prevented • Life-years and quality-adjusted life-years (QALYs) gained • Reduction in OOPE • Number of patients incurring catastrophic and impoverishing health expenditures	Combined secondary and tertiary prevention strategies were most cost-effective. The poorest quartile benefited the most in terms of cases prevented and OOPE reduction.	catastrophic and impoverishing health expenditures	**(Dixit et al.**,** 2023)**
18	Assebe et al. (2020)	Ethiopia	The population at risk of malaria in Ethiopia, which accounts for 60% of the total population.	Population at risk of malaria (2016): 61,504,000 people Population for malaria vaccine (2016 birth cohort): 1,984,000 people	health system	Artemisinin-based combination therapy Long-lasting insecticide-treated bed nets (LLIN) Indoor residual spraying (IRS) Malaria vaccine (hypothetical)	The comparison is made between the existing baseline coverage and the projected coverage after the introduction of universal public financing for the interventions.	• Out-of-pocket outpatient costs • Out-of-pocket inpatient costs • Unit cost of malaria treatment outpatient visit • Unit cost of malaria treatment inpatient visit • Unit cost of LLIN • Unit cost per vaccinated child (3 doses) • IRS unit cost per person protected • Household consumption expenditure • Share of food in total consumption expenditure	1-Deaths averted 2-Private health expenditures eliminated 3-Financial risk protection benefits	The interventions would have the largest impact on malaria-related deaths averted and FRP benefits, especially among the poorest two income quintiles.	In this study, a case of catastrophic health expenditures (CHE) was counted when total OOP spending for malaria treatment exceeded 10% of total household consumption expenditures or 40% of capacity to pay (i.e. non-food total house hold consumption**)**	**(Assebe et al.**,** 2020)**
19	Salti et al. (2016)	Lebanon	50 years	population of smokers	lifetime	50% increase in the price of tobacco through excise taxes	Before and after the tax increase	• Hospitalization cost by tobacco-related disease (2012 USD) • Fraction of healthcare costs paid out-of-pocket by quintile	• Premature deaths averted: 65,000 • Poverty cases averted: 27,000 • Increased tax revenues: $300 million • Healthcare costs averted: $37 million • Out-of-pocket expenditures averted: $22 million	Poorest quintile gains the most health and financial benefits		**(Salti et al.**,** 2016)**

All reported cost values are in U.S. dollars (USD) unless otherwise specified. PPP-adjusted values were extracted when reported in the original studies

### Quality of included studies

The methodological quality of the 19 included studies ranged from moderate to good based on the adapted 20-item CHEC checklist. Most studies adequately described the intervention, comparator, study perspective, and distributional analyses across income groups. However, several methodological items were inconsistently reported. In particular, uncertainty analysis (e.g., probabilistic sensitivity analysis), discounting parameters for costs and health outcomes, and specification of the currency year were frequently unclear or missing. Reporting of incremental results by income quintile was also variable across studies. While these limitations do not invalidate the findings, they indicate heterogeneity in methodological rigor that should be considered when interpreting cross-study comparisons.

### Synthesis of results

This review synthesized evidence from 19 ECEA studies spanning diverse public health interventions and country settings. To highlight consistent patterns in health and financial risk protection (FRP) outcomes, findings were organized by intervention category (Table 2).

**Table 2:** Synthesis of results

Area	Intervention	Key Findings
Vaccination Programs	Rotavirus Vaccination	• reduces childhood morbidity • reduces financial burdens on families(Verguet et al., 2013).
	HPV Vaccination	• Significant long term health benefits in reducing the risk of cervical cancer(Levin et al., 2015).
	Measles Vaccination Strategies	• Evaluates various vaccination strategies, focusing on their impact on children’s health outcomes and social protection. (Driessen et al., 2015).
Taxation Policy	Tobacco Tax	• A beneficial effect on the health of smokers, regardless of their income level(Verguet et al., 2015a). • health improvements and financial protection, especially benefiting vulnerable populations(Postolovska et al., 2018).
	Sugar Sweetened Beverage Tax	• Imposing taxes on sugary drinks supports lower income households by decreasing consumption and enhancing health outcomes(Saxena et al., 2019b).
	Cigarette Tax Increase	• Improved financial protection for low income smokers, along with a reduction in overall smoking rates(Raei et al., 2021).
Maternal and Child Health	Essential Health Interventions	• Improves health outcomes across all income groups. • Showcasing successful investment in maternal and child health(Mao et al., 2023).
Chronic Disease Management	Diabetes Services Uptake Improvement	• Increasing the use of diabetes services among high risk populations(Fraser et al., 2024).
Mental Health Strategies	Ethiopian Mental Health Strategy	• Provides significant health gains and financial protection through strategic funding initiatives(Johansson et al., 2017).
Other Health Interventions	Diarrheal Disease Prevention	• Shielding vulnerable populations from financial risks (Pecenka et al., 2015).
	Malaria Interventions	• health gains • financial risk protection for affected populations(Assebe et al., 2020).
	Rheumatic Fever Control Strategies	• Assesses the effectiveness and fairness of strategies to prevent rheumatic diseases, emphasizing their significant impact on public health(Dixit et al., 2023).
	Cataract Surgery Financing	• Identifies the beneficiaries of financial protection in cataract surgery interventions, with a focus on equity in healthcare access(Essue et al., 2020).

Vaccination programs including rotavirus, HPV, and measles consistently achieved substantial health gains alongside strong FRP effects, particularly among lower-income quintiles. Publicly financed vaccination reduced mortality and out-of-pocket (OOP) expenditures [16, 21].underscoring the dual value of prevention and equity promotion. Fiscal policies, notably tobacco and sugar-sweetened beverage (SSB) taxation, showed marked pro-poor distributional effects. Studies from Iran, Mexico, and Lebanon found that excise taxes improved FRP for poorer households due to higher baseline consumption and greater vulnerability to catastrophic health expenditure (CHE) [17, 26, 27]. These interventions also generated fiscal space for reinvestment in health further discussed in Sect. 4.1. Maternal and child health (MCH) financing reforms demonstrated parallel effects: publicly funded delivery care and service expansion reduced both mortality and OOP payments, with the largest equity gains observed among the poorest groups [21, 24]. Chronic and mental health interventions, though fewer, illustrated the broader applicability of ECEA beyond communicable diseases. Integrated diabetes care and Ethiopia’s National Mental Health Strategy both reduced financial hardship, highlighting underexplored but promising domains for future analyses. Other public health interventions including cataract surgery, rheumatic fever prevention, malaria control, and diarrheal disease treatment similarly revealed that public financing or subsidies yield joint efficiency and equity gains, especially for disadvantaged populations. Across all categories, a consistent message emerged: ECEA enables simultaneous assessment of health outcomes and their distributional impact across income groups, offering decision-makers a nuanced view of both efficiency and fairness. These cross-cutting insights provide the foundation for the interpretive discussion presented in Sect. 4.1.

### Subgroup patterns in financial risk protection (FRP)

To further examine heterogeneity in the distributional impacts of ECEA, we conducted descriptive subgroup comparisons across diverse intervention categories. These analyses synthesized variations in key FRP metrics namely, out-of-pocket (OOP) expenditure reduction, prevention of catastrophic health expenditure (CHE), and mitigation of impoverishment—across preventive interventions (e.g., vaccination programs), fiscal policies (e.g., tobacco and sugar-sweetened beverage taxes), health-financing strategies, and clinical service delivery models.

Our synthesis reveals that **fiscal/taxation policies**,** preventive interventions**,** and health financing policies consistently demonstrate substantial financial risk protection (FRP) benefits**,** particularly for the poorest income quintiles (Q1–Q2)**. This pro-poor pattern is driven by factors such as higher price sensitivity and disease burden among lower-income groups, and the direct removal or reduction of out-of-pocket (OOP) expenditures by these interventions. While all categories show these benefits, specific nuances exist: fiscal policies are noted for being “extremely progressive,” preventive interventions show “strong FRP benefits” and higher cost-effectiveness in lower-income groups, and health financing policies directly remove OOP spending leading to “substantial” and “consistently pro-poor” FRP improvements. These emerging patterns, which highlight the equitable distribution of protective benefits across intervention types, are summarized in Table 3. This analysis does not claim inferential causality but serves to identify patterns that should inform future methodological refinements and policy-oriented research.

**Table 3:** Subgroup comparison of financial risk protection (FRP) outcomes across intervention categories

Intervention Category	Representative Studies	Key FRP Metrics Reported	Population Quintile Most Benefited	Overall FRP Pattern
**1. Fiscal / Taxation Policies** (Tobacco tax, SSB tax)	Verguet 2015 (China), Raei 2021 (Iran), Postolovska 2018 (Armenia), Salti 2016 (Lebanon), Saxena 2019 (SA, PHI), Saenz-de-Miera 2022 (Mexico)	• OOP averted • CHE averted • Poverty cases averted • Money-metric insurance value	Poorest (Q1–Q2)	Extremely progressive; FRP benefits consistently higher in the lowest income groups due to (1) higher price sensitivity, (2) higher disease burden, (3) greater risk of impoverishment.
**2. Preventive Interventions** (Vaccines, vector-control)	Verguet 2013 (Ethiopia/India), Pecenka 2015, Driessen 2015, Assebe 2020	• OOP averted • Private expenditures averted • FRP (insurance value)	Poorest (Q1–Q2)	Strong FRP benefits for the poorest households; cost-effectiveness also higher in low-income groups due to greater disease incidence.
**3. Health Financing Policies** (UPF, vouchers, subsidy schemes)	Verguet 2015b (India TB-UPF), Essue 2020 (Vietnam cataract), Mao 2023 (Nigeria MNCH), Raykar 2016 (India schizophrenia)	• OOP eliminated • CHE averted • Medical impoverishment averted	Poorest (Q1), sometimes middle quintiles	FRP improvements are direct and substantial because interventions remove or reduce OOP spending. Benefits are consistently pro-poor.
**4. Clinical Service Delivery Programs** (Mental health, chronic diseases, maternal care)	Johansson 2017 (Ethiopia), Fraser 2024 (South Africa CCT), Dixit 2023 (India RHD)	• OOP averted • CHE averted • Insurance value	Poorest 40%	FRP benefits notable but vary depending on disease burden and baseline access. Programs with cash transfers show particularly strong FRP effects.

FRP benefits were generally larger for taxation-based interventions compared with preventive programs, particularly in terms of reductions in catastrophic health expenditure and poverty cases averted. Preventive interventions such as vaccination showed more balanced distributional effects across income quintiles, whereas taxation policies demonstrated stronger progressivity, with disproportionately greater FRP gains among lower income groups. These findings are descriptive and reflect patterns observed across heterogeneous study designs.

### Meta-narrative synthesis

To complement the subgroup analyses, a meta-narrative synthesis was conducted to map the distinct research streams within the ECEA literature. This approach allowed us to explore how different bodies of ECEA research conceptualized equity, operationalized Financial Risk Protection (FRP), and applied methodological frameworks across heterogeneous intervention types. The results of this synthesis are presented in Table 4.

**Table 4:** Meta-narrative synthesis: evolution of equity metrics and methodological approaches in ECEA

Row	Research Stream	Conceptualization of Equity	FRP Metrics Used	Methodological Characteristics	Typical Intervention Types	Key Findings / Narrative Themes	Representative Studies
1	Preventive Interventions (Vaccination Programs)	Equity defined mainly through quintile-specific health gains and distribution of mortality averted; some use of concentration indices	OOP averted; limited reporting of CHE or poverty cases	Mostly static or cohort models; societal perspective common; short-to-medium time horizons	Rotavirus, measles, HPV vaccination	Prevention reduces mortality disproportionately in lower-income groups; balanced FRP but strong health gains	Verguet et al., Pecenka et al., Driessen et al.
2	Fiscal and Taxation Policies (Tobacco, SSB Taxes)	Strong equity orientation using progressivity analysis, equity weights, and quintile incidence	OOP averted; CHE averted; poverty cases averted (most comprehensive)	Economic simulation models; lifetime horizon; sensitivity analyses frequently reported	Tobacco taxation, sugar-sweetened beverage taxes	Highest FRP impact among all streams; strongly progressive benefits; large reductions in poverty among bottom quintiles	Verguet et al., Saenz de Miera et al., Raei et al.
3	Health Financing Policies (UPF, Subsidy Models)	Equity defined through income-quintile service coverage and financial burden distribution	CHE averted and poverty cases most common	Mostly societal perspective; long-term horizon; hybrid costing approaches	Universal public financing, maternal–child health financing	Substantial FRP especially in poorest quintiles; reduction of catastrophic payments; expanded utilization	Watkins et al., Raykar et al., Mao et al.
4	Clinical Service Delivery Interventions	Equity measured via differential access and service utilization; few formal equity metrics	Mostly OOP averted; limited CHE reporting	Short-term horizons, facility-based costing; health-system perspective common	Diabetes care, cataract surgery, rheumatic fever programs	Moderate FRP impacts; stronger gains in utilization than financial protection	Caldwell et al., McPake et al.
5	Cross-cutting ECEA Framework Studies	Explicit integration of welfare economics; use of equity weights and distributional welfare indices	All three FRP domains commonly included	Methodological/ conceptual analyses rather than empirical modeling	Framework papers, comparative ECEA	Highlight conceptual gaps, emphasize distributive justice, and strengthen equity-grounded evaluation	Verguet et al. (methodological), Cookson et al.

A meta-narrative synthesis was performed to compare conceptual and methodological streams in the ECEA literature (Table 4).

Meta-narrative comparison of the major research streams within ECEA studies. The table highlights conceptualizations of equity, FRP metrics used, methodological characteristics, intervention typologies, and emergent thematic findings across different streams of the ECEA literature. Findings are descriptive and reflect methodological variability across included studies.

## Discussion

This scoping review mapped the application of Extended Cost-Effectiveness Analysis (ECEA) across a diverse range of health interventions and policy settings and identified important geographic, methodological, and thematic patterns. Although the review applied no geographical restrictions, the available evidence was overwhelmingly concentrated in low- and middle-income countries (LMICs), particularly in Asia and Africa. This finding reflects the historical development of ECEA as an equity-sensitive economic evaluation framework designed to assess not only health gains but also financial risk protection (FRP) and the distribution of benefits across socioeconomic groups. These dimensions are especially relevant in settings where out-of-pocket expenditures remain substantial and where healthcare-related financial hardship continues to be a major policy concern.

Across the included studies, ECEA was most commonly applied to vaccination programs, fiscal policies targeting harmful products, publicly financed health services, and maternal and child health interventions. Despite substantial variation in intervention type and country context, a consistent pattern emerged: health gains and financial protection benefits were disproportionately concentrated among lower-income populations. This finding reinforces the central premise of ECEA that health policies should be evaluated not only according to efficiency criteria but also according to their ability to reduce inequalities and protect vulnerable households from financial hardship [7]. These findings are broadly consistent with the growing literature emphasizing the importance of incorporating equity and financial risk protection considerations into economic evaluation and health priority setting, particularly in the context of Universal Health Coverage.

Preventive interventions, particularly vaccination programs targeting rotavirus, HPV, and measles, represented one of the most prominent applications of ECEA [14, 18]. The reviewed studies consistently demonstrated substantial reductions in mortality alongside significant financial protection benefits. Public financing of vaccination programs reduced household out-of-pocket expenditures, prevented catastrophic health expenditures, and generated the greatest benefits among poorer population groups [12]. These findings are consistent with broader evidence suggesting that preventive interventions can simultaneously improve population health and advance equity objectives. Because many preventive interventions generate benefits over long time horizons, ECEA provides a particularly valuable framework for capturing both immediate and downstream distributional consequences that may be overlooked by conventional cost-effectiveness analyses.

Fiscal policies, especially tobacco and sugar-sweetened beverage taxation, also emerged as a major area of ECEA application. Across multiple country settings, these policies generated substantial health gains and meaningful financial protection effects, particularly among lower-income households [10, 24, 27]. Although consumption taxes are sometimes criticized for their potentially regressive financial effects, the reviewed studies suggest that long-term reductions in healthcare expenditures and disease burden may outweigh short-term economic concerns, especially among populations facing higher risks of tobacco-related illness [15, 19]. These findings illustrate the importance of incorporating distributional and financial protection outcomes into economic evaluations when assessing the overall societal impact of fiscal health policies.

The review further demonstrated that publicly financed health services and maternal and child health programs frequently produced substantial improvements in both health outcomes and FRP indicators. Studies evaluating publicly financed treatment programs for conditions such as tuberculosis and schizophrenia reported important reductions in out-of-pocket expenditures and improvements in insurance value, particularly among disadvantaged socioeconomic groups [14, 17, 18]. Similarly, maternal and child health interventions were associated with meaningful gains in survival and equity outcomes. Collectively, these findings suggest that policies designed to reduce financial barriers to healthcare access may generate benefits that extend beyond health improvement alone and contribute to broader social protection objectives [18, 19].

An important observation from this review is the considerable methodological heterogeneity across ECEA studies. Differences in analytical perspectives, modelling approaches, time horizons, socioeconomic classifications, and definitions of FRP outcomes were common. While this flexibility demonstrates the adaptability of ECEA across diverse policy contexts, it also limits comparability across studies and complicates evidence synthesis. In particular, variation in the measurement of catastrophic health expenditure, poverty cases averted, and out-of-pocket expenditure reductions remains a significant challenge for cross-study comparison. Greater methodological standardization would enhance the consistency, transparency, and policy relevance of future ECEA applications.

The relatively limited number of eligible studies identified in this review further suggests that ECEA remains a developing field of research despite its growing relevance for equity-oriented health policy evaluation.

The geographic distribution of the evidence base also highlights important opportunities for future research. Although ECEA has become increasingly established within LMIC settings, relatively few eligible studies originated from high-income countries (HICs). This imbalance may reflect the traditional emphasis of economic evaluation in HICs on efficiency-based outcomes rather than distributional or financial protection measures. However, persistent inequalities in healthcare access, affordability, and health outcomes remain important concerns across many HICs. Expanding the application of ECEA to areas such as mental health, long-term care, chronic disease management, and preventive health services may therefore provide valuable insights into equity-related consequences that are not routinely captured by conventional economic evaluation methods.

The findings of this review also have important implications for health policy and priority setting. As countries pursue Universal Health Coverage (UHC) and seek to reduce health inequalities, policymakers increasingly require evidence that extends beyond aggregate estimates of cost-effectiveness. By explicitly quantifying the distribution of health gains and financial protection benefits across socioeconomic groups, ECEA offers a framework capable of informing more equitable resource allocation decisions. The consistent pro-poor effects identified across vaccination programs, fiscal policies, and publicly financed services suggest that integrating ECEA into health technology assessment (HTA) and priority-setting processes may support more transparent and equity-sensitive decision-making [13, 19, 23].

Several limitations should be considered when interpreting these findings. The included studies exhibited substantial methodological heterogeneity, which limited direct comparisons across interventions and settings. Variations in modelling assumptions, analytical perspectives, and FRP definitions may have influenced reported outcomes. In addition, several studies relied on secondary expenditure data and modelling assumptions that introduced uncertainty into estimates of financial protection effects. Although no geographic restrictions were applied during study selection, the available evidence remained concentrated in LMICs, limiting the generalizability of findings to other health system contexts. Finally, the exclusion of conference abstracts and unpublished literature may have resulted in the omission of emerging evidence. The relatively limited number of eligible studies identified in this review further highlights that ECEA remains a developing field of research despite its increasing policy relevance.

Overall, the findings indicate that ECEA has emerged as an important framework for evaluating the joint distribution of health and financial outcomes associated with health policies and interventions. Future research should prioritize methodological standardization, broader geographical representation, and the expansion of ECEA applications to underexplored policy domains. Strengthening the evidence base in these areas may further enhance the contribution of ECEA to equity-informed health policy and resource allocation decisions.

## Conclusion

This scoping review mapped the application of Extended Cost-Effectiveness Analysis (ECEA) in health-sector research and identified important geographic and methodological patterns. Although no geographic restrictions were applied, the existing evidence is concentrated primarily in low- and middle-income countries and focuses largely on preventive interventions, taxation policies, and publicly financed health services. Across studies, ECEA was consistently used to evaluate both health gains and financial risk protection across socioeconomic groups.

The review also identified substantial variation in methodological approaches, equity measures, and definitions of financial risk protection, limiting comparability across studies. These findings highlight the need for greater methodological standardization and broader application of ECEA in underrepresented regions and health policy domains. Expanding the use of ECEA may provide additional evidence to support equity-sensitive priority setting and resource allocation decisions in the pursuit of universal health coverage.

### Glossary of key equity terms

- **Financial Risk Protection (FRP)**: Measures the extent to which a health intervention prevents financial hardship, including OOP expenditures averted, catastrophic health expenditure averted, and poverty cases averted.
- **Distributional Impact**: Assessment of how health and financial outcomes are distributed across socioeconomic groups.
- **Equity Weighting**: A method that assigns higher value to health gains among disadvantaged groups.
- **Concentration Index**: A statistical measure of socioeconomic inequality in health outcomes.
- **Income-Quintile Disaggregation**: Reporting outcomes separately for the poorest to richest quintiles.

## Supplementary Information

Below is the link to the electronic supplementary material.

Supplementary Material 1

Supplementary Material 2

Supplementary Material 3

## Data Availability

The datasets generated and analyzed during this scoping review, including the search strategy, study selection criteria, and extracted data, are available from the corresponding author upon reasonable request.
