# Supplementary material for: Extended cost-effectiveness analysis in global health systems: a scoping review of geographic gaps and research trends
Source: Cost Eff Resour Alloc. 2026 Jun 28;24:115. doi: 10.1186/s12962-026-00791-4 (PMC13573463; doi:10.1186/s12962-026-00791-4)
Supplement: Supplementary file 1 [file 12962_2026_791_MOESM1_ESM.docx]

**Consensus Health Economic Criteria (CHEC) checklist**

| **Row** | **Study** | **1** | **2** | **3** | **4** | **5** | **6** | **7** | **8** | **9** | **10** | **11** | **12** | **13** | **14** | **15** | **16** | **17** | **18** | **19** | **20** |
| --- | --- | --- | --- | --- | --- | --- | --- | --- | --- | --- | --- | --- | --- | --- | --- | --- | --- | --- | --- | --- | --- |
| **1** | **Verguet 2013 (India/Eth.)** | Y | Y | Y | Y | Y | Y | Y | U | Y | Y | U | U | U | Y | U | U | U | U | Y | Y |
| **2** | **Verguet 2015 TB (India)** | Y | Y | Y | Y | U | Y | Y | U | Y | Y | U | U | U | Y | U | U | U | U | Y | Y |
| **3** | **Verguet 2015 China (Tobacco)** | Y | Y | Y | Y | Y | Y | Y | U | Y | Y | U | U | U | Y | U | U | U | U | Y | Y |
| **4** | **Saxena 2019 S. Africa (SSB)** | Y | U | U | Y | U | Y | Y | U | Y | Y | U | U | U | Y | U | U | U | U | Y | Y |
| **5** | **Saxena 2019 Philippines** | Y | U | U | Y | U | Y | Y | U | Y | Y | U | U | U | Y | U | U | U | U | Y | Y |
| **6** | **Saenz-de-Miera 2022 (Mexico)** | Y | Y | Y | Y | Y | Y | Y | U | Y | Y | U | Y | U | Y | U | Y | Y | Y | Y | Y |
| **7** | **Raykar 2016 India (Schiz.)** | Y | U | U | Y | Y | Y | Y | U | Y | Y | U | U | U | Y | U | U | U | U | Y | Y |
| **8** | **Raei 2021 Iran (Tobacco)** | Y | Y | Y | Y | Y | Y | Y | U | Y | Y | U | U | U | Y | U | U | U | U | Y | Y |
| **9** | **Postolovska 2018 Armenia** | Y | U | U | Y | U | Y | Y | U | Y | Y | U | U | U | Y | U | U | U | U | Y | Y |
| **10** | **Pecenka 2015 Ethiopia** | Y | U | U | Y | U | Y | Y | U | Y | Y | U | U | U | Y | U | U | U | U | Y | Y |
| **11** | **Mori 2017 Zambia** | Y | Y | Y | Y | U | Y | U | U | Y | Y | U | U | U | Y | U | U | U | U | Y | Y |
| **12** | **Mao 2023 Nigeria (MNCH)** | Y | Y | Y | Y | Y | Y | Y | U | U | Y | U | U | U | Y | U | U | U | U | Y | Y |
| **13** | **Johansson 2016 Ethiopia** | Y | Y | Y | Y | Y | Y | Y | U | Y | Y | U | U | U | Y | U | U | U | U | Y | Y |
| **14** | **Fraser 2024 SA (CCT)** | Y | N | Y | Y | Y | Y | Y | U | Y | Y | U | Y | U | Y | U | Y | Y | Y | Y | Y |
| **15** | **Essue 2019/2020 Vietnam (Cataract)** | Y | Y | Y | Y | U | Y | Y | U | Y | Y | U | U | U | Y | U | U | U | U | Y | Y |
| **16** | **Driessen 2015 Ethiopia (Measles)** | Y | Y | Y | Y | Y | Y | Y | U | Y | Y | U | U | U | Y | U | U | U | U | Y | Y |
| **17** | **Dixit 2023 India (Rheumatic)** | Y | Y | Y | Y | Y | Y | Y | U | Y | Y | U | U | U | Y | U | U | U | U | Y | Y |
| **18** | **Assebe 2020 Ethiopia (Malaria)** | Y | Y | Y | Y | U | Y | Y | U | Y | Y | U | U | U | Y | U | U | U | U | Y | Y |
| **19** | **Salti 2016 Lebanon (Tobacco)** | Y | Y | Y | Y | Y | Y | Y | U | Y | Y | U | U | U | Y | U | U | U | U | Y | Y |
|  | **Notes: Y = Yes, N = No, U = Unclear** | | | | | | | | | | | | | | | | | | | | |
