# Supplementary material for: Extended cost-effectiveness analysis in global health systems: a scoping review of geographic gaps and research trends
Source: Cost Eff Resour Alloc. 2026 Jun 28;24:115. doi: 10.1186/s12962-026-00791-4 (PMC13573463; doi:10.1186/s12962-026-00791-4)
Supplement: Supplementary file 2 [file 12962_2026_791_MOESM2_ESM.docx]

**Search Strategy**

| **Database** | **strategy** | **Results** |
| --- | --- | --- |
| **PubMed** | **1# (“extended cost effectiveness”[Title/Abstract] OR “extended cost-effectiveness”[Title/Abstract] OR ECEA[Title/Abstract] OR “Distributional Cost-Effectiveness Analysis”[Title/Abstract] OR DCEA[Title/Abstract] OR “Equity-Adjusted Cost-Effectiveness Analysis”[Title/Abstract] OR “Comprehensive Cost-Effectiveness Analysis”[Title/Abstract] OR “Augmented Cost-Effectiveness Analysis”[Title/Abstract] OR “Broadened Cost-Effectiveness Framework”[Title/Abstract] OR “Social Cost-Effectiveness Analysis”[Title/Abstract] OR “equity-informative CEA”[Title/Abstract] OR “financial risk protection”[Title/Abstract] OR “poverty impact”[Title/Abstract])**  **2# (“Delivery of Health Care”[Mesh] OR “Health Care Delivery”[Title/Abstract] OR (Delivery[Title/Abstract] AND “Health Care”[Title/Abstract]) OR “Delivery of Healthcare”[Title/Abstract] OR “Healthcare Deliver*”[Title/Abstract] OR (Deliver*[Title/Abstract] AND Healthcare[Title/Abstract]) OR “Health Care System*”[Title/Abstract] OR (System*[Title/Abstract] AND “Health Care”[Title/Abstract]) OR “Healthcare System*”[Title/Abstract] OR (System*[Title/Abstract] AND Healthcare[Title/Abstract]) OR “Health Care”[Title/Abstract] OR (Care[Title/Abstract] AND Health[Title/Abstract]) OR Healthcare[Title/Abstract])**  **1# AND 2#**  **(“extended cost effectiveness”[Title/Abstract] OR “extended cost-effectiveness”[Title/Abstract] OR ECEA[Title/Abstract] OR “Distributional Cost-Effectiveness Analysis”[Title/Abstract] OR DCEA[Title/Abstract] OR “Equity-Adjusted Cost-Effectiveness Analysis”[Title/Abstract] OR “Comprehensive Cost-Effectiveness Analysis”[Title/Abstract] OR “Augmented Cost-Effectiveness Analysis”[Title/Abstract] OR “Broadened Cost-Effectiveness Framework”[Title/Abstract] OR “Social Cost-Effectiveness Analysis”[Title/Abstract] OR “equity-informative CEA”[Title/Abstract] OR “financial risk protection”[Title/Abstract] OR “poverty impact”[Title/Abstract]) AND (“Delivery of Health Care”[Mesh] OR “Health Care Delivery”[Title/Abstract] OR (Delivery[Title/Abstract] AND “Health Care”[Title/Abstract]) OR “Delivery of Healthcare”[Title/Abstract] OR “Healthcare Deliver*”[Title/Abstract] OR (Deliver*[Title/Abstract] AND Healthcare[Title/Abstract]) OR “Health Care System*”[Title/Abstract] OR (System*[Title/Abstract] AND “Health Care”[Title/Abstract]) OR “Healthcare System*”[Title/Abstract] OR (System*[Title/Abstract] AND Healthcare[Title/Abstract]) OR “Health Care”[Title/Abstract] OR (Care[Title/Abstract] AND Health[Title/Abstract]) OR Healthcare[Title/Abstract])** | **547** |
| **Scopus** | **TITLE-ABS-KEY(“extended cost effectiveness” OR “extended cost-effectiveness” OR ECEA OR “Distributional Cost-Effectiveness Analysis” OR DCEA OR “Equity-Adjusted Cost-Effectiveness Analysis” OR “Comprehensive Cost-Effectiveness Analysis” OR “Augmented Cost-Effectiveness Analysis” OR “Broadened Cost-Effectiveness Framework” OR “Social Cost-Effectiveness Analysis” OR “equity-informative CEA” OR “financial risk protection” OR “poverty impact”) AND TITLE-ABS-KEY(“Health Care Delivery” OR (Delivery AND “Health Care”) OR “Delivery of Healthcare” OR “Healthcare Deliver*” OR (Deliver* AND Healthcare) OR “Health Care System*” OR (System* AND “Health Care”) OR “Healthcare System*” OR (System* AND Healthcare) OR “Health Care” OR (Care AND Health) OR Healthcare)** | **680** |
| **Web of Science** | **TS=(“extended cost effectiveness” OR “extended cost-effectiveness” OR ECEA OR “Distributional Cost-Effectiveness Analysis” OR DCEA OR “Equity-Adjusted Cost-Effectiveness Analysis” OR “Comprehensive Cost-Effectiveness Analysis” OR “Augmented Cost-Effectiveness Analysis” OR “Broadened Cost-Effectiveness Framework” OR “Social Cost-Effectiveness Analysis” OR “equity-informative CEA” OR “financial risk protection” OR “poverty impact”) AND TS=(“Health Care Delivery” OR (Delivery AND “Health Care”) OR “Delivery of Healthcare” OR “Healthcare Deliver*” OR (Deliver* AND Healthcare) OR “Health Care System*” OR (System* AND “Health Care”) OR “Healthcare System*” OR (System* AND Healthcare) OR “Health Care” OR (Care AND Health) OR Healthcare)** | **473** |
| **ProQuest** | **TI,AB,SU(“extended cost effectiveness” OR “extended cost-effectiveness” OR ECEA OR “Distributional Cost-Effectiveness Analysis” OR DCEA OR “Equity-Adjusted Cost-Effectiveness Analysis” OR “Comprehensive Cost-Effectiveness Analysis” OR “Augmented Cost-Effectiveness Analysis” OR “Broadened Cost-Effectiveness Framework” OR “Social Cost-Effectiveness Analysis” OR “equity-informative CEA” OR “financial risk protection” OR “poverty impact”) AND TI,AB,SU(“Health Care Delivery” OR (Delivery AND “Health Care”) OR “Delivery of Healthcare” OR “Healthcare Deliver*” OR (Deliver* AND Healthcare) OR “Health Care System*” OR (System* AND “Health Care”) OR “Healthcare System*” OR (System* AND Healthcare) OR “Health Care” OR (Care AND Health) OR Healthcare)** | **255** |
| **total** | **Updated search in 29 October 2025**  **Duplicated=1158**  **Records after deleting duplicates=797** | **1955** |
