# Supplementary material for: Extended cost-effectiveness analysis in global health systems: a scoping review of geographic gaps and research trends
Source: Cost Eff Resour Alloc. 2026 Jun 28;24:115. doi: 10.1186/s12962-026-00791-4 (PMC13573463; doi:10.1186/s12962-026-00791-4)
Supplement: Supplementary file 3 [file 12962_2026_791_MOESM3_ESM.docx]

**Table S1**

| **Study** | **FRP Calculation Method** |
| --- | --- |
| **Fraser et al. (2024, South Africa – diabetes services)** | **OOP reduction only; no CHE or poverty threshold reported → Partially specified** |
| **Pillarisetti et al. (2017 – household energy, India)** | **CHE defined as OOP >10% total annual consumption; poverty line used: national poverty line** |
| **Verguet et al. (2016/2015 – tobacco tax China)** | **CHE threshold 10% of consumption; poverty cases using international poverty line $1.25/day** |
| **Watkins et al. (2017 – cardiovascular risk)** | **OOP reduction only; no explicit CHE or poverty threshold → Not fully specified** |
| **Levin et al. (2015 – HPV, China)** | **CHE threshold 10%; poverty cases: $1.25/day (international poverty line)** |
| **Raei et al. (2021 – Iran cigarette tax)** | **CHE threshold 10% of income; poverty line: national Iranian poverty line** |
| **Saxena et al. (2019 – SSB Philippines)** | **OOP reduction estimated; CHE threshold not defined → Not specified** |
| **Saxena et al. (2019 – SSB South Africa)** | **CHE threshold 10%; poverty cases estimated using national poverty line** |
| **Saenz-de-Miera et al. (2022 – tobacco Mexico)** | **CHE threshold 10%; poverty cases using national poverty line** |
| **Verguet et al. (2013 – rotavirus India & Ethiopia)** | **CHE ≥10% of consumption; poverty cases: $1.25/day** |
| **Dixit et al. (2023 – RHD India)** | **CHE defined as 10% consumption; poverty cases using $1.90/day** |
| **Verguet et al. (2015 – TB India)** | **CHE ≥10% consumption; poverty line $1.25/day** |
| **Raykar et al. (2016 – schizophrenia India)** | **OOP reduction only; CHE/pov thresholds not reported → Not specified** |
| **Driessen et al. (2015 – measles Ethiopia)** | **CHE ≥10% consumption; poverty cases: $1.25/day** |
| **Pecenka et al. (2015 – diarrheal treatment Ethiopia)** | **CHE ≥10%; poverty: $1.25/day (international poverty line)** |
| **Johansson et al. (2017 – Ethiopia mental health)** | **OOP reduction only; CHE/pov methods not reported → Not specified** |
| **Assebe et al. (2020 – malaria Ethiopia)** | **CHE threshold 10%; poverty defined at $1.90/day** |
| **Enden et al. (2021 – modern contraception Ethiopia)** | **OOP reduction only; FRP definition not provided → Not specified** |
| **Essue et al. (2020 – cataract Vietnam)** | **CHE ≥10% of consumption; poverty threshold (national)** |
| **Salti et al. (2016 – tobacco Lebanon)** | **CHE defined at 10% total expenditure; poverty measured by national poverty line** |
| **Mao et al. (2023 – MNCH Nigeria)** | **CHE ≥10% consumption; poverty cases using national poverty line** |
